# The Evolution of Cold Adaptation Technology within Ancient Buildings in Amur River Basin Viewed from Archaeology

**DOI:** 10.3390/ijerph192114470

**Published:** 2022-11-04

**Authors:** Wenqing Liu

**Affiliations:** College of Engineering, Shantou University, Shantou 515063, China; wqliu@stu.edu.cn

**Keywords:** ancient architecture in amur river basin, insulation technology, archaeology, architecture conservation

## Abstract

The Amur River Basin is located in China’s high-latitude and cold border areas. While inheriting the characteristics of Chinese traditional building, combined with its unique geographical and climatic environmental conditions, the local residential buildings have unique architectural features of cold climate adaptability. Outstanding insulation technology has become the main feature of the area, and has greatly affected the development of modern vernacular architecture. According to the archaeological reports and documents, this article selects ancient architectural sites from different historical periods as the research objects. Based on building restoration, AIRPAK software is used to simulate and analyze the indoor temperature of the building site, and to explore the effects of active heating measures, such as different forms of fire hypocaust system, and passive cold protection measures, such as different types of wall structures. According to archaeological information and simulation data, this paper summarizes the characteristics of the cold climate adaptability technology of ancient buildings in China’s cold border areas over different historical periods. Because of the relatively lagging development background of the Amur River Basin in modern times, the construction of its vernacular buildings continued to use the traditional low-tech insulation technology of ancient buildings to adapt to the cold environment. Therefore, attention and research on insulation technology of ancient buildings can provide a new perspective of architectural heritage protection in cold regions. Establishing a development model that combines archaeology and cultural heritage protection is an effective way to achieve the goals of architectural cultural heritage research and protection.

## 1. Technical Features of Building Cold Climate Adaptability

The climate adaptability of ancient settlement and vernacular building is an important root of architecture regionalism [1], it could be a positive model of sustainable development in terms of energy consumption, environmental impact, and carbon emissions [2], and has become a kind of sustainable approach for architecture [3,4]. In recent decades, climate adaptability of ancient buildings has been a rising concern for the energy-saving [5], thermal comfort [6,7], and architectural heritage protection [8,9]. Based on the classification of many typical vernacular buildings in different climate regions, the energy consumption simulation is taken and compared with the modern thermal model, the results show that the materials and forms of most ancient vernacular buildings have a good performance for energy saving [10]. Ancient vernacular building has strong adaptability to the climate environment, and passive heating and cooling measures play an important role in this. In different climate regions from −10 °C to 40 °C around the world, there are diverse ancient buildings that do not rely on fossil energy to meet the needs of human habitation [11]. Appropriate building materials, building envelope, and building shape are important passive factors of comfortable thermal environment and building energy saving [12,13]; these measures are widely used in vernacular architecture of cold regions, together with active heating technology, to cope with the cold climate.

As the tenth largest river basin in the world, the Amur River Basin (41–56 north latitude, and 107–142 east longitude) is situated in northeastern Asia with a relatively high average latitude. Because of its unique geographical and climate environment, in the long process of adapting to the low temperature climate in cold winters, ancient human settlements have made remarkable achievements in terms of building cold adaptability. From the macro perspective, human settlements of cold regions, such as urban area and rural settlements, will always be located reasonably by combining them with the geographical environment, and then strive for favorable natural conditions such as a good orientation and solar radiation, etc. For example, the community of the Sanjiang Plain has an obvious tendency to distribute gentle slopes toward the sun. Central cities are widely distributed in the basin and plains, and a relatively closed geographic space is chosen to avoid the invasion of winter wind fields [14]. Similarly, each urban residential unit makes full use of spatial layout, reasonable material selection, and other measures to achieve spatial integrity (reduce the shape coefficient of a building) and maintain the thermal inertia of the building, thereby achieving adjustments to cold climates. From the micro perspective, each building unit is more inclined to adopt active heating measures such as hypocaust to achieve a comfortable indoor thermal environment.

## 2. Passive Insulation Technology of Building Enclosure Structure

The exterior wall is an important part of the building envelope and the most important loadbearing and heat transfer component in residential buildings, and its material and thickness has a particularly important impact on the service life, heating energy consumption, safety, and comfort of the building in cold areas [15]. As the main body of the outer envelope, the outer wall is exposed to the external environment all the time, and accounts for the largest proportion of the heat loss of the entire building envelope. Therefore, the material and construction of the exterior wall are particularly critical.

### 2.1. The Material Construction Method

Judging from the archaeological information of buildings in the Amur river basin, typical ancient building walls mainly include stone masonry walls, wood-mixed-grass mud wall, rammed-earth walls, brick walls, etc. The exterior walls of the building are the main load-bearing parts, and the wall thickness varies from 300–600 cm without a special thermal insulation layer, only lime mortar and a mixture of mud and grass are applied to the outer layer for heat preservation. According to the archaeological excavation, there are few building sites with clear wall remains, and these building sites include the simple tent camp, small semi-crypt buildings, large semi-crypt buildings, and large ground buildings, as well as typical cases from the Stone Age to the advanced Iron Age, which are shown in Table 1.

From the early Stone Age to when the semi-crypt house sites were created, small temporary camps used plant material such as canvas for wall-like maintenance structures. Branches and grasses were overlapped to form a ventilated and cool environment in the summer, while in the winter, animal skins were used to keep warm. There was a raised boss at the junction of the canvas and the ground, which is convenient to prevent the penetration of cold wind [16,23]. From the Neolithic Age to the Early Iron Age, in the prevalent semi-crypt house sites, the cave wall and wood-mixed-grass mud roof were the main envelope structures of buildings. The main indoor active surface was below ground level, and the appropriate depth of ground reduction was to avoid cold winds. The effective method was also conducive to the internal heat preservation of the house. In the Balhae Kingdom period (698–926), the walls of large wooden frame ground buildings were mainly made of rammed earth, including wood–bone plate building walls, wood–bone adobe walls, and adobe masonry walls [24]. The thermal insulation performance of rammed earth walls was usually better than that of simple wood–bone mud walls and masonry walls, and it was more conducive to forming a stable and suitable indoor temperature. In the Liao and Jin Dynasties (907–1234), the ground house sites and the walls of large-scale timber-framed buildings were pillar-clad brick walls and blue brick masonry walls, and were compared with civil–timber composite walls. The large-scale application of masonry bricks has reduced the insulation ability of the wall maintenance structure, which has led to the phenomenon of excessive wall thickness. The thickness of the brick wood composite wall is more than 0.6 m [20], while the thickness of blue brick masonry wall in some important architectural sites is even more than 1.3 m [22]. From what we have seen so far in the archaeology of ARB, most of the wall remains are incomplete and mainly belong to three periods, as shown in Table 2.

From the early simple tent canvas structure in the Yanjiagang site to the complex blue brick masonry wall in the western building site of the Jin shangjing city site [16,21], the wall construction technology of ancient buildings in the ARB has been constantly changing and progressing. Meeting the requirements of structure and function, the performance of cold protection and heat preservation has always been an important consideration for maintaining the structure. Judging from the archaeological information of the current wall remains, there are two main ways to maintain wall insulation. One is to use the inherent physical properties of the material to form a good insulation effect, such as animal skin curtains and rammed earth walls [16,17,18]. The other is to use the construction methods to enhance the thermal insulation effect, such as increasing the thickness of the wall and the adoption of a composite wall structure made up of different material layers [19,20,21,22]. The gradual development of wall construction technology in different periods reflects outstanding technological progress, but at the same time it is necessary to think about whether the thermal performance of building walls has also been gradually improved with the improvement of construction technology. Therefore, it is particularly necessary to analyze the thermal parameters of wall materials.

### 2.2. The Thermal Parameters of Wall Materials

From the early Stone Age to the Liao and Jin periods, based on archaeological reports, the main building materials used to maintain the structure in each period were used as the research objects to analyze the physical properties of different materials. The thermal conductivity, heat transfer resistance, heat transfer coefficient, and other indicators are used as a reference index, and the specific structure data in the archaeological report are used for the calculation to analyze and compare the effects of different building materials and construction methods on the thermal performance of building walls in various historical periods. According to “the code for thermal design of civil building of China (GB50176-2016)”, and by summarizing the main building materials in different periods, we could calculate the corresponding thermal parameters of each material (Table 3).

The thermal conductivity of animal skin leather is 0.18, and the thickness is generally 0.003 m when used as a building envelope, and the thermal resistance is calculated to be 0.017. Using the same method to calculate other materials, the thermal resistance is 0.1, 0.19, 0.37, 0.39, 0.5, and 1.0, respectively. 

The total thermal resistance of the structure needs to be calculated according to the actual situation. Paleolithic buildings are generally made of grass branches and animal skins. The total thermal resistance of the maintenance structure is the sum of the two layers of construction materials, which is 0.25 after calculation. During the Han and Wei dynasties, semi-crypt house sites were mainly used. The building maintenance structure consists of three layers: 0.28 m of rammed earth, 0.05 m of yellow mud for filling joints, and 0.2–0.75 m of stone masonry. When the wall thickness was 0.25 m, the total thermal resistance was 0.61. The total thermal resistance was 1.05 when the thickness was 0.8 m. During the Balhea period, wood-frame walls consisted of three layers: double 0.15 m cement plastered surface and 0.15 m diameter wooden pillars. The total thermal resistance was 0.71. During the Liao and Jin Dynasties, the buildings were dominated by brick exterior walls. They consisted of three layers: two layers of 0.15–0.20 m lime mortar and 0.3–0.9 m blue bricks. When the wall thickness was 0.6 m, the result was 0.59; when the wall thickness was 1.3 m, it was 1.26.

Equation (1):

K = 1/Ro. w/(m k) Ro = Ri + R + Re. m k/w(1)

Ro is the heat transfer resistance of envelope (m^2^ k/w), Ri is the inner surface heat transfer resistance (m^2^ k/w), Re is the outer surface heat transfer resistance(m^2^ k/w), and K is the heat transfer coefficient (m^2^ k/w).

Through the above calculation, the heat transfer resistance of each material layer is obtained, and the heat transfer resistance and heat transfer coefficient of walls are calculated according to Equation (1), as shown in Table 4.

According to “the General code for energy efficiency and renewable energy application in buildings (GB50015-2021)” and other regulations, when the average outdoor temperature in cold areas is below 11 °C, the heat transfer coefficient limit of the building envelope is 0.52, when the shape coefficient of a building is S ≤ 0.3, and when the building shape coefficient S > 0.3, the heat transfer coefficient limit value is 0.40. According to Table 4, the building of the ancient human settlements exceeded the limit value and do not meet the energy conservation standards.

The thermal resistance of a single material from animal skins to bricks increased from 0.017 to 0.5. This shows that as the selection and use of ancient building materials tend to be rigorous, building materials with a larger thermal resistance are selected to better meet the requirements. However, it can be seen from the data of the heat transfer coefficient of the building envelope that with the advancement of building technology and the update of materials, the cold weather adaptability has not been improved all the time, and there has even been a short-term lag in development.

With the development of building structural technology and social progress, in order to make up for the inadequacy of passive cold protection and heating measures, in addition to passive heating measures in terms of site selection, building structure, and materials, ancient humans developed active heating measures such as hypocausts, fire walls, and chimneys. Through the effective combination of active heating and passive heat preservation, it can meet the requirements of human housing for warmth and improve the comfort of living.

## 3. Active Heating Measures

The emergence of new measures to prevent cold in buildings marks the beginning of the transition from passive heat preservation to active heating in ancient buildings of the ARB, which further increased the indoor temperature in winter and improved the overall performance of the thermal insulation.

### 3.1. Evolution of Hypocaust Types

The hypocaust system is a kind of widely used central heating device in buildings for coping with cold weather. In ancient Rome, hypocaust was often integrated with roofs, floors, and walls for heating the building space [25]. In ancient Korea, a similar heating device is used called ondol, which generates high temperature flue gas to heat the ground by burning fuelwood. Now, ondol is becoming a heating method suitable for modern living forms, and has begun to be used in high-rise residential buildings [26]. In ancient China, a similar device is used called fire ground or fire kang, and it is the most common indoor heating measure in rural areas of northeast China. However, it is still a highly controversial question as to when and where the fire kang originated. Ancient books of the “Compilation of the Three Northern Alliances” of the Song dynasty (960–1279) record it as “the room is surrounded by a soil bed, the lower part is burning, the bedroom living on the top”, there is an image description of the hypocaust called “the soil bed”, which means that it is constructed of masonry, soil, and other materials in the room above the ground. The heat of the flue gas flows in the flue below to achieve the effect of indoor heating. Before the advent of the hypocaust, ancient indoor heating methods were scorched ground. Large tracts of red-burned soil surfaces appeared in the house ruins at the Banpo site in Xi’an, indicating that the heating technology of the barbecue floor appeared in the Neolithic period, and it is generally believed that in the Han Dynasty (BC202-220) at the latest, fire heating ground appeared in the buildings of northern China [27]. With the emergence of farming and settlement patterns during the Han and Wei Dynasties, hypocaust began to appear in the building sites of the ARB as a fixed heating measure. Judging from the remains in the architectural sites of different periods, the forms of the hypocaust of the ARB are complex and diverse, and with the gradual development and advancement of structural technology, the structural styles of the hypocaust show an increasingly delicate development trend.

Therefore, the classification of hypocaust is extremely critical. It can be classified from two aspects: the number of flues and the plane form. According to the number of flues, it can be divided into single flue type, double flue type, three flue type, and multiple flues type; according to the plane shape, it can be divided into curved-foot type, rectangular type, “п” type, and whole surface type (Table 5).

We used several sites as examples to analyze the evolution of the hypocaust system. The earliest hypocaust remains appeared in the lower building site of Dongning Site and Fenglin Site in the Eastern Han Dynasty, and these sites were mostly single-strand chimneys without vertical chimneys. The hypocaust system was connected by the stove site to the flue, and the heating efficiency was very low. The construction method and system structure of the Balhae Kingdomhave made great progress. The remains at the Longquan Mansion Palace Site show that the strand flue evolved into multiple strand flue. During the Liao and Jin Dynasties, it further evolved into multiple flues and vertical chimneys. The information of specific hypocaust sites for three periods is shown below (Table 6). Therefore, it is concluded that the evolution process of the hypocaust progressed from simple to complex. The specific performance was the change from single-strand to double-strand and multiple strands in the flue. The plane form changed from I-shape to L-shape, п -shape, and more complicated styles. Smoke holes evolved to vertical chimneys higher than the surface, and the construction materials changed from simple materials to adobe masonry. The progress of building technology promotes the development of hypocaust heating technology, but the effect of heating measures on the indoor temperature of ancient buildings still needs further simulation and calculation.

### 3.2. The Indoor Temperature Simulation of Ancient Buildings with Hypocaust

We used the evolution of the hypocaust as a reference, selected three typical examples, and used Airpak for building restoration and thermal performance simulation. F10 in Fenglin Site [18], Palace No.4 site (sleeping hall) in Shangjing city site of Balhae state [19], and F6 in Lantoubao site [20] were chosen to study the cases of heating on hypocaust in different periods (Table 7).

**Figure 1:**
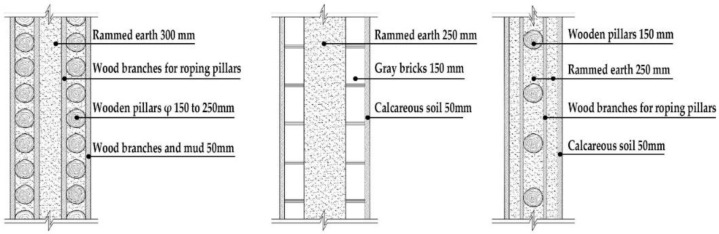
The construction of material layers of exterior walls for three models (from left to right, cross section of F10 in the Fenglin site, F6 in the Lantoubao site, and Palace 4 in the Shangjing city sites).

Based on the archaeological excavation information of three relics and the meteorological information from the China Meteorological Information Center, the space models and heat transfer models could be built in Airpak software. Firstly, the material type and thickness of the enclosure interface of the three building relics were selected and built in their space models. For the walls, the materials and thickness were specified according to Table 1, and details of their material construction are shown in Figure 1. For the interior floor, the material construction of sleeping hall No. 4 palace was a single layer of blue brick with rammed earth cushion [19], while the floors of F6 and F10 were both rammed earth covered with a hard surface [18,20]. For the roofs, pitched wooden roof of three buildings were converted to a flat wooden roof with equal thickness for the feasibility of modeling and computation. 

Then, the heat transfer models were established through optimizing space models and parameter settings. Site locations of the three relics were specified according to their longitude and latitude coordinates, and the simulated date was set as 20 January which is usually the coldest day of a year. The month with the lowest average temperature in ARB is January, according to the dataset of daily surface climatological data for China (V3.0, National Meteorological Information Center of China), the average minimum temperature in January from 1999 to 2020 of the weather stations near the three relics ranged from −18 to −22 °C mostly, and −18 °C, −20 °C, and −22 °C were chosen as the ambient temperatures of the sleeping hall, F6, and F10, respectively. 

The surface temperature of the kang is an important model parameter, in some research about ondol in cold areas of China, the geometric model of kang is often simplified to a uniform heating surface [34,35], but the temperature difference between the head part and the end part of the kang can be up to 20 °C [36], so the heating surface should be divided into several parts, and then the temperature distribution of each part is assumed to be uniform [37]. For reasons of accuracy, the hypocaust surfaces of the sleeping hall, F6, and F10 were divided into two or three parts with different (detail) temperatures.

**Figure 2:**
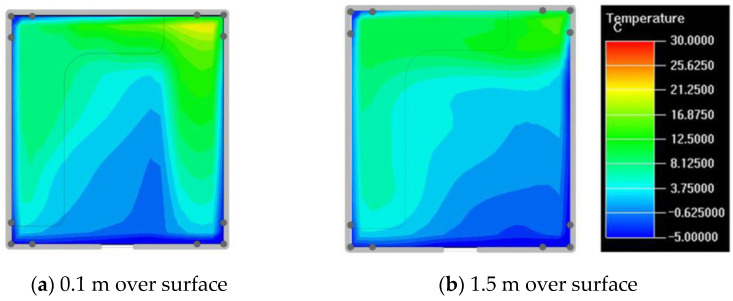
The indoor temperature of F10.

**Figure 3:**
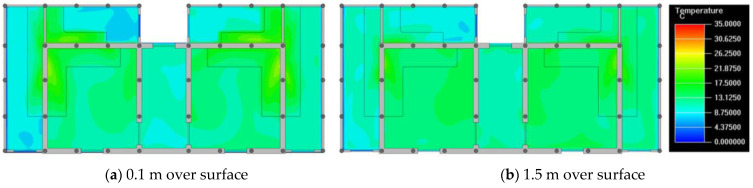
The indoor temperature of No. 4 Palace.

**Figure 4:**
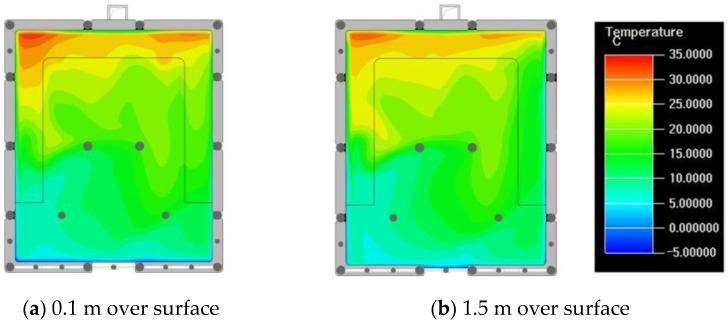
The indoor temperature of F6.

Finally, based on the worst cold conditions, the temperature distribution cloud map of the section 0.1 m and 1.5 m above the surface of the fire hypocaust in each building site was obtained through simulation calculations (Figure 2, Figure 3 and Figure 4).

During the Han and Wei periods, the hypocaust was a single flue and L-shaped, with an indoor area of about 17 square meters. The average indoor temperature was mostly concentrated in the range of 2–15 °C and the main uniformity of temperature distribution was insufficient, and the low temperature area is mostly below 0 °C. In the Balhae Kingdom period, the hypocaust was a compound type with multiple flues, and the indoor area was about 300 square meters. The average temperature was mostly concentrated in the range of 10–22 °C, and the main living space had no obvious low temperature area. The average temperature of the side chamber was low, but the overall temperature distribution was relatively balanced. During the Liao-Jin period, the hypocaust was a multiple flue and was U-shaped, with an indoor area of 165 square meters. The average indoor temperature was concentrated in the range of 12–25 °C, and there was no obvious low temperature area. There was a high temperature aggregation phenomenon near the north surface of the hypocaust, and the overall temperature distribution was average.

Generally speaking, from the Han and Wei periods to the Liao and Jin periods, the heating performance of the hypocaust in the house gradually improved. The indoor temperature simulation results show that the average temperature of the cross-section of the main activity height gradually increased, and the adaptability of residential buildings to the cold climate gradually strengthened. For small houses such as Fenglin F10, the unbalanced distribution of the indoor temperature was serious; for medium-sized houses like Lantoupu F6, the indoor high temperature areas were relatively concentrated. The temperature distribution of the No.4 palace was more even, there were no over-cooling or over-heating areas, and the indoor environment was relatively comfortable.

## 4. Discussion

The archaeological research on the thermal insulation measures of ancient buildings in ARB shows that the cold adaptability of vernacular buildings can be guaranteed by a hypocaust system and appropriate building enclosure materials, and it has two implications on modern building heating technology.

On the one hand, research on passive energy-saving technology for rural houses in the cold areas of north China is being explored in recent years, and a series of rural residential projects have been constructed [38,39,40], but the poor thermal performance of the enclosure surface of modern rural residential buildings makes the indoor temperature easily affected by outdoor temperature changes, when the outdoor temperature reaches −30 °C, the indoor temperature drops below 10 °C in severe cold regions, which seriously affects the indoor thermal comfort [41]. The cold-adaptation techniques of ancient buildings provide a valuable model for selecting or inventing the material structure of external walls and other enclosure surfaces. At the same time, winter heating consumption dominated by fossil fuels and electricity has increased dramatically in rural buildings of north China, the proportion of biofuels in energy consumption is decreasing [42], and a large-scale survey of rural buildings in 24 provinces shows that high energy consumption and poor indoor environment are important challenges for the sustainable development of rural buildings [43,44]. Thus, a low-carbon and sustainable cold climate adaptation method is urgently needed, and the traditional hypocaust system in ancient buildings of ARB is an appropriate method to learn from, especially for the energy-saving optimization of countless rural houses in severe cold regions.

On the other hand, the history of cold climate adaptability of ancient buildings in ARB has evolved for thousands of years. It is not only a technical measure, but also a part of architecture and cultural heritage. The passive insulation technology reflected in the building forms and enclosure interfaces is one of the roots of the regional characteristics of modern vernacular buildings in cold areas, and the active heating system of the hypocaust can not only ensure thermal comfort, but also a place for sleeping and activities, and a symbol of traditional rural civilization that cannot be abandoned [45]. In the conservation process of vernacular architectural heritage, cold climate adaptability should not be ignored.

## 5. Conclusions

In summary, the cold climate adaptability of buildings in ABR reflects the wisdom and creativity of the ancient civilizations. Despite the relatively lagging development in modern times, the low-tech forms of ancient architecture can still be used and improved continuously, and they are still the main cold-protection method for vernacular buildings. The use of primitive technology to guide modern building can expand the new perspective of the protection of architectural heritage in cold regions for the attention and research of cold protection technology in ancient buildings. Through the establishment of a development model that combines archaeology and cultural heritage protection, this is an effective way to achieve the goals of architectural cultural heritage research and protection.

## Figures and Tables

**Table 1:** The types, examples, and material structures of exterior walls in Amur river basin [16,17,18,19,20,21,22].

Building Sites	Wall Type	Wall Structure
The Round House in Yanjiagang —Old Stone Age [16]	Tent curtain wall	Temporary curtain structure made of branches and leaves.
Yinggeling Upper Remains F1—Neolithic age [17]	Stone masonry wall	Masonry stone walls filled with mud, 0.25 m thickness.
Fenglin Ancient City Remains F23—Early Iron Age [18]	Wood-mixed-grass mud wall	The wall thickness is 0.35–0.65 m, and the diameter of the wooden bone column is 0.15–0.4 m.
Balhae kingdom Palace No.4 Remains—Mature Iron Age [19]	Timber plate-building wall	Black soil, 0.3–0.4 m thickness, with wooden pillars diameter 0.15 m.
Jin Dynasty Dehui Ruins F6—Developed Iron Age [20]	Pillar-clad brick walls	The wall is about 0.6 m thick, single-built gray bricks, filled with soil.
Western Building Site of Jinshangjing City—Developed Iron Age [21]	Blue brick masonry walls	Blue brick masonry wall, 0.9 m thickness, with sand and mud plastering on the outside.
Ruins of Changbaishan Temple—Jin Dynasty- Developed Iron Age [22]	Brick masonry walls	The wall thickness is about 1.3 m, the blue brick masonry methods are various.

**Table 2:** The examples of material remains of walls in three periods [18,19,20,22].

Period	Typical Remain Examples			
Wall materials remains in Hanwei period (the Early Iron Age of ARB)	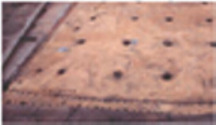		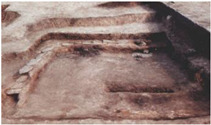	
	F10 and F34 of Fenglin City Site [18]			
Wall materials remains of Bohea kingdom (698–926)	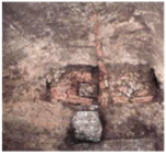	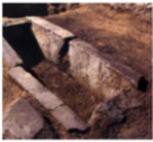		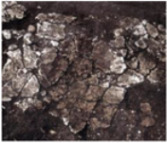
	The Palace 2 of Longquan City Site [19]			
Wall materials remains in Liaojin Dynasties (907–1234)	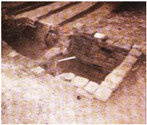	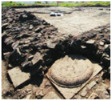 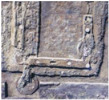		
	F6 of Lantou Site [20]	F3 and F4 of Changbai Temple Site [22]		

**Table 3:** Building material thermal coefficient index.

Material	Thermal Conductivity	Thermal Effusivity
Hay	0.05 (W/m·K)	0.83 (W/(m^2^·k))
leather	0.18 (W/m·K)	14.74 (W/(m^2^·k))
Rammed soil	0.76 (W/m·K)	9.37 (W/(m^2^·k))
Wood (horizontal)	0.17 (W/m·K)	4.9 (W/(m^2^·k))
Wood (vertical)	0.38 (W/m·K)	6.93 (W/(m^2^·k))
Sand lime brick masonry	1.10 (W/m·K)	12.72 (W/(m^2^·k))

**Table 4:** Thermal performance index of the envelope building structure.

Wall Structure	R (K/W)	Ro (K/W)	K (W/m^2^·K)
tent canvas	0.25 (K/W)	0.4 (K/W)	2.5 (W/m^2^·K)
Stone masonry (d = 0.25 m)	0.61 (K/W)	0.7 (K/W)	1.32 (W/m^2^·K)
Stone masonry (d = 0.8 m)	1.05 (K/W)	1.2 (K/W)	0.8 (W/m^2^·K)
wooden rammed earth	0.71 (K/W)	0.8 (K/W)	1.169 (W/m^2^·K)
blue brick masonry (d = 0.25 m)	0.59 (K/W)	0.7 (K/W)	1.359 (W/m^2^·K)
blue brick masonry (d = 1.3 m)	1.26 (K/W)	1.4 (K/W)	0.71 (W/m^2^·K)

**Table 5:** Hypocaust shape classification.

Flues and Plane Form	Single Flue	Double Flue	Three and Multiple Flues
curved-foot type;	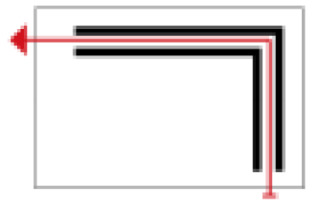	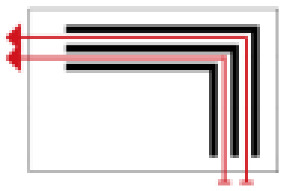	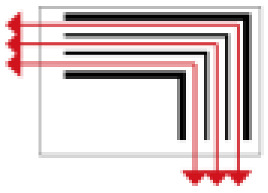
	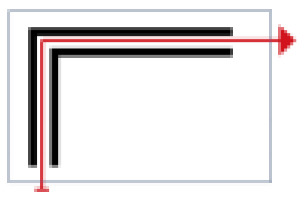	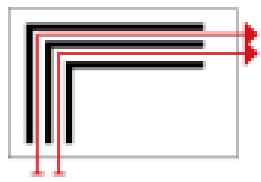	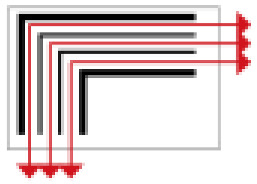
rectangular type	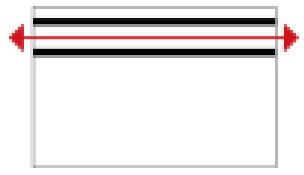	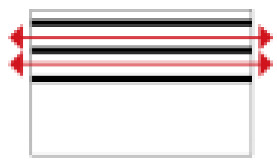	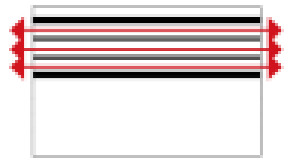
	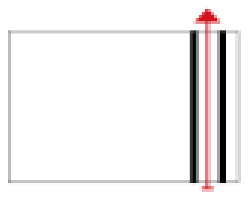	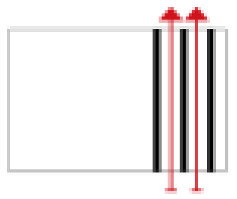	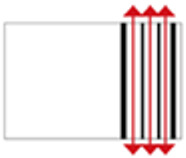
“п” type	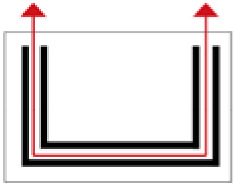	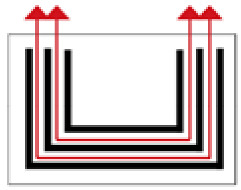	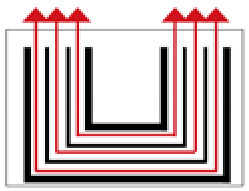
whole surface type	-	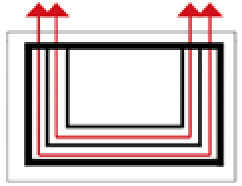	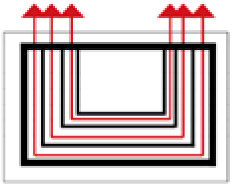

**Table 6:** The heated kang relics of different periods in the Amur river basin [28,29,30,31,32,33].

Type	Remain Examples		
Single flue heated Kang of Hanwei period	** 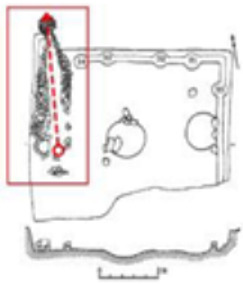 **	** 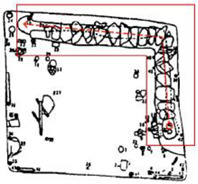 **	** 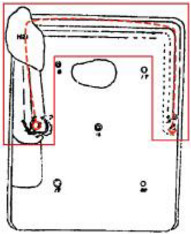 **
	Dongning Xiaodiying Site F1 [28]	F10, Fenglin City Site [18],	F9 of Dongning Tuanjie Site [29]
Double and multiple flue heated Kang of Bohea kingdom	** 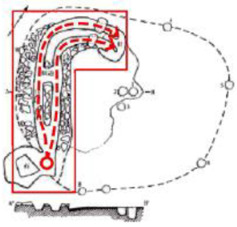 **	** 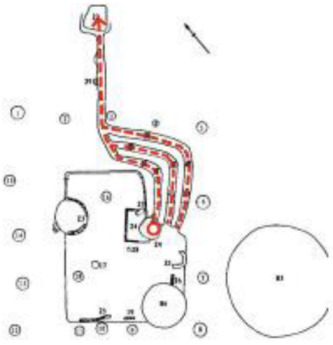 **	** 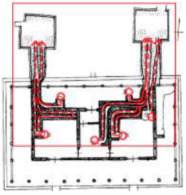 **
	Hailin Xingnong City Site F3 [30]	Suibin Tongren Site F1 [31]	Longquan Mansion Palace [19]
Multiple flue heated Kang of Bohea kingdom	** 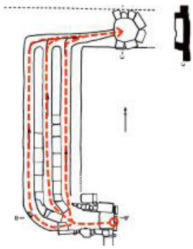 **	** 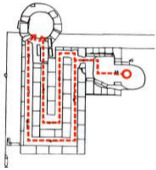 **	** 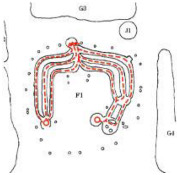 **
	Yongping Liaojin Site F4 [32]	Yongping Liaojin Site F1 [32]	Lishu Bakeshu Site F1 [33]

**Table 7:** Case site plan and model diagram [18,19,20].

Building Sites	Fenglin F10 of Hanwei Period	No. 4 Palace of Bohea Capital	Lantoubao F6 of Jin Dynasty
Site plan	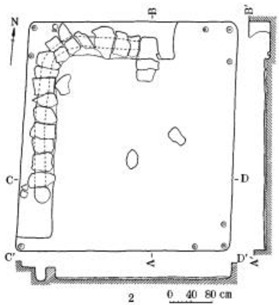	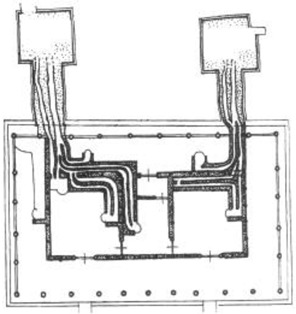	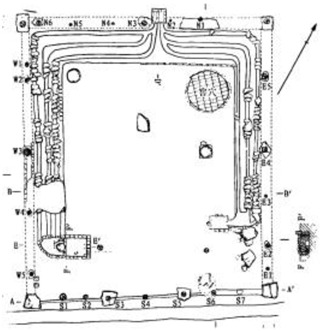
Model diagram in AIRPAK	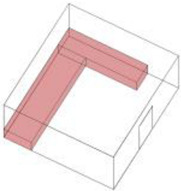	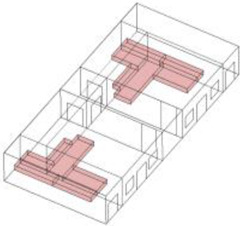	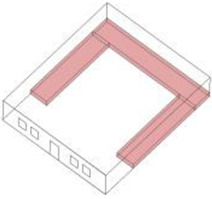

## Data Availability

The data used in this study are available from the corresponding author on reasonable request.
